# Association of Monocyte-to-HDL Cholesterol Ratio with Endothelial Dysfunction in Patients with Type 2 Diabetes

**DOI:** 10.1155/2024/5287580

**Published:** 2024-01-11

**Authors:** Huihui Zhang, Jun Lu, Jie Gao, Wenjun Sha, Xinhua Cai, Mai Re Yan Mu Rouzi, Yuanying Xu, Wenjun Tang, Tao Lei

**Affiliations:** ^1^School of Medical and Life Sciences, Chengdu University of Traditional Chinese Medicine, Chengdu, China; ^2^Department of Endocrinology, Putuo Hospital, Shanghai University of Traditional Chinese Medicine, Shanghai, China; ^3^Heart Function Examination Room, Tongji Hospital, Tongji University, Shanghai, China

## Abstract

**Aims:**

To explore the relationship between monocyte-to-HDL cholesterol ratio (MHR) and endothelial function in patients with type 2 diabetes (T2DM).

**Methods:**

243 patients diagnosed with T2DM were enrolled in this cross-sectional study. Patients were divided into two groups by flow-mediated dilation (FMD) quintile as nonendothelial dysfunction (FMD ≥ 6.4%) and endothelial dysfunction (FMD < 6.4%). The relationship between MHR and FMD was analyzed using Spearman's correlation, partial correlation, and multiple logistic regression analysis. ROC curve was fitted to evaluate the ability of MHR to predict endothelial dysfunction.

**Results:**

Endothelial dysfunction was present in 193 (79%) patients. Patients with endothelial dysfunction had higher MHR (*p* < 0.05) than those without endothelial dysfunction. Furthermore, MHR had a significantly positive correlation with endothelial dysfunction (*r* = 0.17, *p* < 0.05), and the positive association persisted even after controlling for confounding factors (*r* = 0.14, *p* < 0.05). Logistic regression showed that MHR was an independent contributor for endothelial dysfunction (OR: 1.35 (1.08, 1.70), *p* < 0.05) and the risk of endothelial dysfunction increases by 61% with each standard deviation increase in MHR (OR: 1.61 (1.12, 2.30), *p* < 0.05) (model 1). After adjusting for sex, age, BMI, disease course, hypertension, smoking, and drinking (model 2) as well as HbA1c, HOMA-IR, C-reactive protein, and TG (model 3), similar results were obtained. In ROC analysis, the area of under the ROC curve (AUC) for MHR was 0.60 (95% CI 0.52-0.69, *p* < 0.05).

**Conclusion:**

MHR was independently associated with endothelial dysfunction in T2DM patients. It could be a new biomarker for vascular endothelial function assessment.

## 1. Introduction

Diabetic angiopathy is the most devastating complication of diabetes, including micro- and macrovascular diseases which are characterized by abnormal growth and repair dysfunction of blood vessels [1]. At the early stage of diabetic angiopathy, the vascular endothelial damage is a key event contributing to the progressive loss of vascular repair mechanisms [2]. Flow-mediated dilation (FMD) is a tool for examining the dysfunction of vascular endothelial cells. FMD represents an endothelium-dependent, largely nitric oxide- (NO-) mediated dilatation of conduit arteries in response to an imposed increase in blood flow and shear stress and possibly identifying subjects at increased risk for future cardiovascular events [3]. It was reported that the risk of cardiovascular adverse event will increase by 13% with the FMD decreases by 1% [4]. Recently, FMD was used to screen and monitor the progression of endothelial dysfunction in diabetic patients by physicians.

Several studies have shown that the vascular endothelial cells were exposed to high level of glucose developing lesions through multiple signaling pathways, including pathways associated with impaired endothelial functions [5–8] and inflammatory pathways [9–12]. Indeed, many studies have suggested that endothelial dysfunction was mainly resulted from inflammation, especially mediated by monocytes and macrophages [13, 14]. After being produced and released into peripheral circulation by the bone marrow hematopoietic system, monocytes interact with endothelial cells to coordinate inflammation, angiogenesis, and tissue remodeling [15]. Adhesion of circulating monocytes to endothelial cells is a crucial step in the pathogenesis of inflammation and atherosclerosis. It was reported that intercellular cell adhesion molecule-1 (ICAM-1) and vascular cell adhesion molecule-1 (VCAM-1) could promote the adhesion of human monocytic THP-1 cell to HUVECs, which accelerated the development of atherosclerosis [16]. On the other hand, epidemiological studies have suggested an inverse correlation between high-density lipoprotein cholesterol (HDL-C) levels and the risk of cardiovascular disease [17]. High-density lipoproteins (HDLs) are involved in a pivotal metabolic pathway that excess cholesterol from peripheral tissues is transported to the liver for excretion in the bile, which is so-called “reverse cholesterol transport.” Reverse cholesterol transport from macrophages in atherosclerotic plaques is a critical mechanism of the antiatherogenicity of HDLs [18–22]. Besides, HDLs decrease the expression of adhesion molecules and upregulate endothelial nitric oxide synthase (eNOS) expression, improving the endothelial function [23]. Therefore, the rate between monocytes and HDL-C can reflect the inflammatory status and associate with endothelial dysfunction.

To the best of our knowledge, there was no study to investigate the association of MHR with FMD in type 2 diabetes. As mentioned above, we postulated that the integrated biomarker MHR was associated with FMD. Hence, we aimed to evaluate the relationship between MHR and endothelial dysfunction in this study.

## 2. Materials and Methods

### 2.1. Study Design and Subjects

This cross-sectional study was conducted in a single center at Putuo Hospital, which was affiliated to the Shanghai University of Traditional Chinese Medicine. 243 participants presented with DM-related symptoms such as excessive thirst or blurred vision between September 2022 and March 2023 underwent FMD. Individuals with type 1 diabetes, ketoacidosis, infectious disease, hematological diseases, rheumatic diseases, and malignant tumors and for whom there were no blood routine examination and liver function test records when the study was conducted were excluded. Finally, 133 patients participated in this study. The FMD of endothelial dysfunction patients were under 6.4% (*n* = 193), whereas controls were not lower than 6.4% (*n* = 50) (Table 1).

The study was approved by the institutional review board of Shanghai University of Traditional Chinese Medicine's Putuo Hospital and was conducted according to the principles articulated in the second version of the Helsinki Declaration. All participants provided written informed consent, and their individual information was kept strictly confidential.

### 2.2. Anthropometric and Laboratory Measurement

The patient's anthropometric and biochemical measurements were taken at admission. The height and weight were measured to calculate the body mass index (BMI) by using the formula weight/height^2^ (kg/m^2^) [24] while the patients were barefoot and in light clothing. The blood pressure was measured twice after the patient rested about 10 minutes and calculated the average value. The fasting blood samples were taken for measurement of fasting insulin, fasting plasma glucose, lipid profiles, WBC, and monocyte automatically using a Beckman Coulter AU5800 biochemical analyzer (Brea, CA, USA) [24]. HOMA-IR was calculated by using the formula fasting plasma glucose multiplied by fasting insulin and then divided by 22.5. MHR was determined by dividing the monocyte counts (in 10^9^/l) by HDL-C (in mmol/l). HbA1c was measured by high-performance liquid chromatography on a Tosoh Automated Glycohemoglobin Analyzer HLC-723G11 (Shunan, Yamaguchi, Japan) [24].

### 2.3. Definitions of Hypertension and Type 2 Diabetes Mellitus

Hypertension was defined as blood pressure (BP) ≥ 140/90 mmHg or usage of antihypertensive medications [25]. Type 2 diabetes mellitus was defined as classic diabetes symptoms with a random plasma glucose ≥ 200 mg/dl (11.1 mmol/l), or FPG ≥ 126 mg/dl (7.0 mmol/l), or 2 h PG ≥ 200 mg/dl (11.1 mmol/l) during OGTT, or HbA1c ≥ 6.5%, and the diagnosis is needed to be rechecked another day as there were no typical diabetes symptoms [26].

### 2.4. Assessment of Brachial Artery Flow-Mediated Dilatation

Endothelium-dependent FMD in response to reactive hyperemia of the brachial artery were evaluated using a high-resolution B-mode ultrasonographic system (Yikangda AGE pro, Anhui, China) and performed by the same technician in a darkened and quiet room which the temperature was about 25°C. All patients were evaluated between 09 : 00 and 11 : 00 am after abstaining from alcohol, caffeine, and tobacco and fasting for 12 hours. Vessel base diameter and vessel dilatation diameter of all patients were recorded after a resting period of 10 minutes. The FMD was calculated by using the formula (vessel dilatation diameter − vessel base diameter)/vessel base diameter × 100%. The patients were divided into two groups based on the FMD according to quintiles: nonendothelial dysfunction as FMD ≥ 6.4% and endothelial function as FMD < 6.4%.

### 2.5. Statistical Analysis

Statistical analysis was performed by using the SPSS for Windows 26.0 software. The Kolmogorov–Smirnov test was used to assess the distribution of all continuous variables, and those conforming normal distribution were described by $\bar{x}\pm s$. Non-normally distributed continuous variables were expressed as *M* (*Q*_L_, *Q*_U_) and the categorical variables as *n* (%). Differences between groups were examined by the Mann–Whitney *U* test for continuous variables and chi-square test for categorical variables. Spearman's correlation and multivariable logistic regression were utilized to analyze the association between FMD and MHR. ROC curve was used to assess the sensitivity and specificity of MHR's ability to predict endothelial dysfunction.

## 3. Results

### 3.1. Clinical Characteristics of Patients Included

Table 1 shows the basic clinical characteristics of patients finally included. 193 patients had endothelial dysfunction in this study. The subjects in the endothelial dysfunction group were obviously older than those in nonendothelial dysfunction (66.6 vs. 63, *p* = 0.004). Patients with endothelial dysfunction had higher SBP (135 vs. 130, *p* = 0.001) and DBP (81 vs. 80, *p* = 0.012) compared to patients without endothelial dysfunction. The MHR level of endothelial dysfunction patients was significantly higher than the group of nonendothelial dysfunction (0.40 (0.30-0.50) vs. 0.40 (0.30, 0.40), *p* = 0.009). Those with endothelial dysfunction had significantly higher monocyte counts (0.45 vs. 0.37, *p* = 0.002) than those with nonendothelial dysfunction. There was no statistically significant difference between the study groups in terms of sex, smoking, drinking, BMI, disease course, hypertension, FBG, FCP, HbA1c, TC, TG, LDL-C, HDL-C, and WBC levels.

### 3.2. Correlation of Endothelial Dysfunction with Other Indicators

Table 2 expresses the association of endothelial dysfunction with MHR and other indicators. Spearman's correlation showed that endothelial dysfunction had a remarkably positive association with MHR (*r* = 0.17, *p* = 0.009). In addition, endothelial dysfunction was positively correlated with age (*r* = 0.18, *p* = 0.004), SBP (*r* = 0.22, *p* = 0.001), DBP (*r* = 0.16, *p* = 0.012), and drinking (*r* = 0.14, *p* = 0.034). We found that the indicators MHR (*r*′ = 0.14, *p* = 0.042), SBP (*r*′ = 0.24, *p* < 0.001), and DBP (*r*′ = 0.22, *p* = 0.001) still remained the positive association with endothelial dysfunction after controlling the variables sex and age, despite the coefficient of association of MHR was decreased. However, the association between endothelial dysfunction and drinking (*r*′ = 0.09, *p* = 0.19) was disappeared following the two variables fixed.

### 3.3. Association of MHR Levels with Endothelial Dysfunction

It was identified that MHR is an independent risk factor for endothelial dysfunction in patients with T2DM. As shown in Table 3, for every 10 percent increase in MHR was positively correlated with endothelial dysfunction in unadjusted model (model 1) (crude odds ratio 1.35, 95% confidence interval (CI) 1.08-1.70), after adjusting for sex, age, BMI, disease course, hypertension, smoking, and drinking (OR 1.34, CI 1.04-1.73), as well as further adjustment for HbA1c, HOMA-IR, C-reactive protein, and TG (OR 1.35, 95% CI 1.02-1.77). The risk of endothelial dysfunction was increased layer by layer from the lowest MHR level tertile to the highest. In comparison with tertile 1, ORs in tertile 2 and tertile 3 were 1.29 (95% CI 0.64-2.62) and 3.62 (95% CI 1.51-8.68) in model 1, respectively. The corresponding ORs were 1.07 (95% CI 0.50-2.27) and 3.70 (95% CI 1.4-9.85) in adjusted model 2 and 0.94 (95% CI 0.42-2.12) and 3.50 (95% CI 1.24-9.93) in adjusted model 3, respectively (all *p* for trend < 0.05). Each one standard deviation (SD) increase in MHR (10^9^/mmol) was associated with a 1.61-fold (95% CI 1.12-2.30) increase of endothelial dysfunction risk in model 1. After adjustment for sex, age, BMI, disease course, hypertension, smoking, drinking, HbA1c, HOMA-IR, C-reactive protein, and TG, similar results were obtained (OR 1.59, 95% CI 1.06-2.38, model 2; OR 1.6, 95% CI 1.04-2.46, model 3).

### 3.4. Receiver Operating Characteristic Curves of MHR for Predicting Endothelial Dysfunction

**Figure 1:**
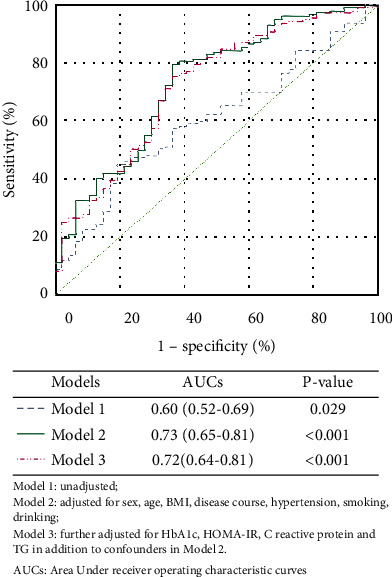
Receiver operating characteristic curves for predicting endothelial dysfunction. Model 1: unadjusted; model 2: adjusted for sex, age, BMI, disease course, hypertension, smoking, and drinking; model 3: further adjusted for HbA1c, fasting C-peptide, and TG in addition to confounders in model 2; AUCs: area under curves.

According to the models of regression analysis, ROC curves (Figure 1.) were fitted to investigate the ability of MHR in predicting endothelial dysfunction in type 2 diabetes patients. Data showed that with fixed variables, sex, age, BMI, disease course, hypertension, smoking, and drinking, in model 2, the area under the curve (AUC) increased when compared to model 1 (0.73 vs. 0.60, *p* < 0.05) and model 3 (0.73 vs. 0.72, *p* > 0.05) and the AUC in model 3 was smaller than model 2 which further adjusted HbA1c, HOMA-IR, C-reactive protein, and TG.

## 4. Discussion

Diabetic angiopathy is the main cause for diabetic-related death and disability [27]. It was reported that diabetes was responsible for 1.5 million deaths which almost half of those occurring before the age of 70. Additionally, high blood sugar contributed to approximately one-fifth of all cardiovascular disease deaths [28]. The damage of vascular endothelial cells is a crucial event at the early stage of diabetic angiopathy. Endothelial cells, as a barrier between vessel wall and blood, are the basis of vascular function. Endothelial dysfunction was described by reduced production of nitric oxide (NO) which is associated with vasodilatory function and increased receptors of the endothelial surface for leukocytes. It is easy for leukocytes to enter into endothelial subintimal layer after being combined with these receptors and gradually develop to the early stages of atherosclerosis. Our assessment for vascular endothelial function-FMD can evaluate the vascular function based on the endogenous vasodilation function of the endothelium, which can better reflect the true physiological state of the blood vessels. In addition, it is easier to detect the endothelial function in clinical due to its noninvasive advantages.

In the present study, T2DM patients with vascular endothelium dysfunction tended to have higher SBP and DBP, which was agreed with previous studies [29, 30]. To a certain extent, blood pressure can reflect the functional status of vascular endothelial. One of the physiological functions of vascular endothelial cells is to regulate vascular tone and maintain normal blood pressure. Besides, the population of endothelial dysfunction subjects with T2DM was older than the nonendothelial dysfunction group. Aging has been identified as a major risk factor for vascular disease [31].

The major finding in our investigation was that the relationship between MHR and endothelial dysfunction was positive significantly. The MHR was obviously higher in the group of endothelial dysfunction compared to the normal group. Multiple regression analysis showed that MHR was an independent risk factor for endothelial dysfunction. With every 10% increase in MHR, the risk of triggering endothelial dysfunction increases by 35%, and the risk of endothelial dysfunction increases by 61% with each standard deviation increase in MHR.

Our data showed that monocyte level in endothelial dysfunction with T2DM patients elevated compared to nonendothelial dysfunction subjects. The majority of T2DM patients are always accompanied by obesity which can lead to insulin resistance and subsequently impair beta cells. The impairment of islet beta cells can trigger systemic inflammation and metabolic syndrome, ultimately resulting in leukocyte activation [32, 33]. Excessive activation of leukocytes is prone to endothelial injury, which accelerates the process of atherosclerosis [34]. There were several studies that described the association between monocyte and the function of vascular endothelial. From a life-adult study, the data showed that monocyte subtype counts were associated with 10-year cardiovascular disease risk as determined by the Framingham risk score, and monocyte counts could provide useful predictive value for cardiovascular disease risk [35]. Recently, another research team found that monocyte cell counts could predict the occurrence of macrovascular complications in patients with diabetes mellitus [36]. These reports well reflected the close relationship between monocytes and endothelial function, which were consistent with our findings.

Besides, type 2 diabetes patients are at a high risk for dyslipidemia, including elevated triglycerides, increased low-density lipoprotein cholesterol, and decreased HDL-C [37]. The characteristics of HDL-C protecting endothelium were described as counteracting LDL oxidation, decreasing the expression of endothelial surface adhesion factor, and preventing the apoptosis of endothelium [38, 39]. The latest study found that the reconstituted HDL-apoE3 significantly enhanced endothelial cell migration in vitro and improved vascular permeability in vivo, which could promote re-endothelialization and retard development of atherosclerosis [40]. Consequently, the deficiency of HDL-C is associated with endothelial dysfunction and a key contributor to promote atherosclerosis in diabetic patients. A report revealed that MHR was associated with carotid intima-media thickness (CIMT) and suggested that MHR was a convenient and effective measure in prediction of the presence and progression of subclinical carotid atherosclerosis in patients with T2DM [30]. Our results indicated that MHR was closely related to FMD and a good indicator of endothelial function.

There are some limitations in our investigation. Firstly, this study had a small sample size and was a cross-sectional study in a single center. Secondly, some medications with an effect on vascular endothelial function, such as GLP-1, SGLT-2, ACEI, and CCB, were missing in this study. Thirdly, serum factors that reflect endothelial function, such as endothelial microparticles (EMPs), endothelin nitric oxide synthase (eNOS), and endothelin-1 (ET-1), were not tested.

## 5. Conclusion

In summary, this report showed that elevated MHR was a convenient and effective measurement in prediction of the occurrence of endothelium impairment and could be a novel index to value the endothelial function in patients with T2DM.

## Figures and Tables

**Table 1:** Clinical characteristics of patients included.

	All (*n* = 243)	Nonendothelial dysfunction (*n* = 50)	Endothelial dysfunction (*n* = 193)	*p* value
Men (%)	146 (60.1)	26.0 (52.0)	120 (62.0)	0.35
Age (years)	65.7 (59.6-72.4)	63 (54.6-67)	66.6 (60-73)	0.004
Smoking (%)	60 (24.7)	9 (18.0)	51 (26)	0.21
Drinking (%)	61 (25.1)	7 (14.0)	54 (28)	0.10
BMI (kg/m^2^)	24.2 (22.4-26.3)	24.2 (22.1-26.2)	24.4 (22.5-26.4)	0.48
Disease course (%)	10.0 (3.00-20.0)	10.0 (2.00-16.0)	10.0 (4.00-20.0)	0.30
SBP (mmHg)	137 ± 0.17	129 ± 2.17	139 ± 1.33	0.001
DBP (mmHg)	83 ± 0.67	80 ± 1.39	84 ± 0.75	0.012
Hypertension (%)	147 (60.5)	25 (50.0)	122 (63)	0.09
FBG (mmol/l)	7.70 (5.90-10.2)	7.85 (5.93-10.2)	7.60 (5.85-10.2)	0.89
HbA1c (%)	9.6 (7.9-11.2)	9.2 (7.9-11.2)	9.7 (7.9-11.3)	0.46
Fasting C-peptide(mmol/l)	0.35 (0.19-0.60)	0.34 (0.16-0.60)	0.35 (0.19-0.60)	0.57
HOMA-IR	26.2 (14.4-48.1)	19.6 (10.6-51.6)	27.3 (14.9-47.1)	0.44
TC (mmol/l)	4.59 (3.72-5.56)	4.75 (3.80-5.57)	4.51 (3.71-5.56)	0.82
TG (mmol/l)	1.42 (0.99-2.05)	1.37 (0.99-2.06)	1.43 (1.00-2.06)	0.52
HDL-C (mmol/l)	1.07 (0.94-1.26)	1.05 (0.94-1.37)	1.07 (0.95-1.26)	0.78
LDL-C (mmol/l)	2.95 (2.3-3.62)	2.89 (2.42-3.61)	2.96 (2.26-3.63)	0.89
WBC (10^9^/l)	6 (4.9-7.1)	5.5 (4.9-6.4)	6.1 (4.9-7.2)	0.12
Monocyte (10^9^/l)	0.43 (0.35-0.54)	0.37 (0.31-0.50)	0.45 (0.37-0.55)	0.002
MHR	0.43 ± 0.01	0.37 ± 0.02	0.44 ± 0.01	0.009
FMD (%)	4.45 ± 0.13	7.57 ± 0.12	3.65 ± 0.10	<0.001

Data are presented as median (IQR) for continuous variables and number (proportion%) for category variables. Differences in medians were examined using the Mann–Whitney *U* test between two groups. Differences in proportions were tested using the chi-square test. BMI: body mass index; SBP: systolic blood pressure; DBP: diastolic blood pressure; FPG: fasting plasma glucose; TC: total cholesterol; TG: triglyceride; HDL-C: high-density lipoprotein cholesterol; LDL-C: low-density lipoprotein cholesterol; WBC: white blood cell; MHR: monocyte-to-high-density lipoprotein cholesterol ratio.

**Table 2:** Correlation of endothelial dysfunction with other indicators.

Variables	Spearman's correlation		Partial correlation	
	*r*	*p* value	*r*	*p* value
Sex (male)	-0.09	0.160	—	—
Age (years)	0.18	0.004	—	—
BMI (kg/m^2^)	0.05	0.481	0.10	0.127
Disease course (years)	0.07	0.300	0.02	0.806
SBP (mmHg)	0.22	0.001	0.24	<0.001
DBP (mmHg)	0.16	0.012	0.22	0.001
Smoking (%)	0.08	0.209	0.07	0.334
Drinking (%)	0.14	0.034	0.09	0.190
HbA1c (%)	0.05	0.465	0.09	0.164
HOMA-IR	0.05	0.436	-0.02	0.749
TC (mmol/l)	0.02	0.816	0.12	0.068
TG (mmol/l)	0.04	0.517	0.11	0.098
HDL-C (mmol/l)	-0.02	0.783	-0.04	0.511
LDL-C (mmol/l)	0.01	0.889	0.09	0.211
MHR	0.17	0.009	0.14	0.042

BMI: body mass index; SBP: systolic blood pressure; DBP: diastolic blood pressure; TC: total cholesterol; TG: triglyceride; HDL-C: high-density lipoprotein cholesterol; LDL-C: low-density lipoprotein cholesterol; MHR: monocyte-to-high-density lipoprotein cholesterol ratio.

**Table 3:** Association of MHR levels with endothelial dysfunction.

	Model 1		Model 2		Model 3	
	Crude OR (95% CI)	*p* value	OR (95% CI)	*p* value	OR (95% CI)	*p* value
MHR per 0.1	1.35 (1.08-1.70)	0.010	1.34 (1.04-1.73)	0.026	1.35 (1.02-1.77)	0.033
MHR tertiles						
Tertile 1	1 (REF)	—	1 (REF)	—	1 (REF)	—
Tertile 2	1.29 (0.64-2.62)	0.47	1.07 (0.50-2.27)	0.86	0.94 (0.42-2.12)	0.89
Tertile 3	3.62 (1.51-8.68)	0.004	3.70 (1.40-9.85)	0.009	3.50 (1.24-9.93)	0.020
*p* trend	1.80 (1.20-2.70)	0.004	1.76 (1.13-2.75)	0.013	1.70 (1.06-2.73)	0.027
MHR per SD	1.61 (1.12-2.30)	0.010	1.59 (1.06-2.38)	0.026	1.60 (1.04-2.46)	0.033

Model 1: unadjusted; model 2: adjusted for sex, age, BMI, disease course, hypertension, smoking, and drinking; model 3: further adjusted for HbA1c, HOMA-IR, C-reactive protein, and TG in addition to confounders in model 2; OR: odds ratio; 95% CI: 95 % confidence interval.

## Data Availability

The original data used in this study are available from the corresponding authors upon request.

## Abbreviations

T2DM: Type 2 diabetes
HDL-c: High-density lipoprotein cholesterol
HDLs: High-density lipoproteins
MHR: Monocyte-to-HDL cholesterol ratio
FMD: Flow-mediated dilation
AUC: Area under receiver operating characteristic (ROC) curves
EMPs: Endothelial microparticles
ET-1: Endothelin-1
eNOS: Endothelin nitric oxide synthase
NO: Nitric oxide
VCAM-1: Vascular cell adhesion molecule-1
ICAM-1: Intercellular cell adhesion molecule-1.
